# Complete Lipopolysaccharide of *Piscirickettsia salmonis* Is Required for Full Virulence in the Intraperitoneally Challenged Atlantic Salmon, *Salmo salar*, Model

**DOI:** 10.3389/fcimb.2022.845661

**Published:** 2022-03-18

**Authors:** Valeska Herrera, Nicole Olavarría, José Saavedra, Yassef Yuivar, Patricio Bustos, Oscar Almarza, Marcos Mancilla

**Affiliations:** ^1^ Blue Genomics SpA, Puerto Varas, Chile; ^2^ Laboratorio de Diagnóstico y Biotecnología, R & D Department, ADL Diagnostic Chile, Puerto Montt, Chile

**Keywords:** *Piscirickettsia salmonis*, piscirickettsiosis, lipopolysaccharide, virulence, Salmo salar, SRS, biofilm

## Abstract

Bacterial cell envelopes play a critical role in host-pathogen interactions. Macromolecular components of these structures have been closely linked to the virulence of pathogens. *Piscirickettsia salmonis* is a relevant salmonid pathogen with a worldwide distribution. This bacterium is the etiological agent of piscirickettsiosis, a septicemic disease that causes a high economic burden, especially for the Chilean salmon farming industry. Although *P. salmonis* has been discovered long ago, its pathogenicity and virulence mechanisms are not completely understood. In this work, we present a genetic approach for producing in-frame deletion mutants on genes related to the biosynthesis of membrane-associated polysaccharides. We provide a detailed *in vitro* phenotype description of knock-out mutants on *wzx* and *wcaJ* genes, which encode predicted lipopolysaccharide (LPS) flippase and undecaprenyl-phosphate glucose phosphotransferase enzymes, respectively. We exhibit evidence that the *wzx* mutant strain carries a defect in the probably most external LPS moiety, while the *wcaJ* mutant proved to be highly susceptible to the bactericidal action of serum but retained the ability of biofilm production. Beyond that, we demonstrate that the deletion of *wzx*, but not *wcaJ*, impairs the virulence of *P. salmonis* in an intraperitoneally infected Atlantic salmon, *Salmo salar*, model of piscirickettsiosis. Our findings support a role for LPS in the virulence of *P. salmonis* during the onset of piscirickettsiosis.

## Introduction

*Piscirickettsia salmonis* is a Gram-negative bacterium of the class *Gammaproteobacteria* and an intracellular facultative microorganism that causes piscirickettsiosis, a deadly and highly contagious disease of reared salmonids (Fryer and Hedrick, 2003). Although the pathogen is globally disseminated, virulent strains are particularly prevalent in the south of Chile where frequent outbreaks occur that often need to be controlled by antimicrobial therapy (Rozas and Enriquez, 2014). Piscirickettsiosis is also a big concern for salmon producers from an economic point of view: the annual costs of mortality, vaccines and antibiotic treatments may add up to 700 million dollars, according to the Technological Institute of Salmon, Intesal (https://www.intesal.cl/). Due to its environmental dimension, the required amount of antibiotics is also a regulatory and trading issue. Therefore, addressing this sanitary threat is a priority for several stakeholders in the Chilean industry.

In recent years, it has become evident that *P. salmonis* shares some features found in other, better-known pathogens. In the context of its genetic relationship to *Legionella*, a functional effector export machinery, including type 4 secretion systems or T4SS, was found to be expressed *in vitro* and required to survive *in vivo* (Gomez et al., 2013; Mancilla et al., 2018). In this regard, adhesins such as T4SS pilin have been demonstrated to occur *in vitro* (Sánchez et al., 2018). Flagellar proteins have been detected within *P. salmonis* cells, but motility has not been described (Carril et al., 2016). *P. salmonis* seems to require iron, since the bacterium expresses siderophores, and salmonids respond to the infection using iron-deprivation as a defense mechanism (Pulgar et al., 2015; Almarza et al., 2016; Calquin et al., 2017). More recently, gene expression studies have accounted for the expression of putative virulence factors carried by cryptic plasmids, as well as several chromosomal and plasmid-borne antibiotic resistance markers (Sandoval et al., 2016; Saavedra et al., 2018; Ortiz-Severin et al., 2020). Altogether, it becomes clear that the intracellular phase of the *P. salmonis* life cycle requires adaptive traits for survival within macrophages. Bactericidal mechanisms, for instance, maybe overcome by interference with intracellular vesicle traffic (Perez-Stuardo et al., 2019; Zuniga et al., 2020). The ability of biofilm formation has also been explored, and the transition from planktonic growth to cells embedded in an exopolysaccharide (EPS) matrix has been postulated as an adaptive life form needed for survival under stress conditions in the marine environment (Marshall et al., 2012; Albornoz et al., 2017).

Gram-negative bacteria share a common cell envelope structure, with a peptidoglycan polymer interspaced between a double lipid bilayer. The outer membrane is mainly composed of an amphipathic chemical species called lipopolysaccharide (LPS), a complex macromolecule containing three canonical moieties: lipid A is anchored to the membrane and connects to the outermost motif known as O-antigen or O-chain *via* the core oligosaccharide (Samuel and Reeves, 2003). The bacterial membrane can also be covered by a matrix of polysaccharide nature derived from the export of EPS. If this polysaccharide remains attached to the surface, it is referred to as capsule (Reid and Szymanski, 2010). Otherwise, this polymer may also be enzymatically released and contribute to biofilm formation (Donot et al., 2012). In bacterial pathogens, it is well established that both polysaccharides LPS and EPS are critical for the maintenance of cell integrity, adhesion, bacteriophage receptors, resilience to host defense mechanisms, recognition by the host immune system, and virulence (Moradali and Rehm, 2020). Attempts to gain insight into the structure and function of LPS and EPS have been reported (Vadovic et al., 2007; Marshall et al., 2012; Vinogradov et al., 2013), but the role of these biopolymers in the pathogenicity of *P. salmonis* remains unclear.

Since our understanding of the pathogen’s biology and virulence mechanisms will benefit the development of effective disease prevention and management strategies, the goal of this study was to shed light on the role of cell envelope polysaccharides in the virulence of *P. salmonis*. We provide genetic and functional evidence on the identification of a gene cluster encoding biosynthetic functions for both LPS and EPS within the genome of *P. salmonis*. To further support our hypothesis, we generated *P. salmonis* mutants defective in LPS or EPS using a genetic approach designed to overcome unwanted side effects and to facilitate the study of their virulent behavior *in vitro* and *in vivo*.

## Material and Methods

### Bacterial Strains, Plasmids, and Culture Conditions

Bacterial strains and plasmids used throughout this study are listed in **Table 1**. P*. salmonis* strains were cultivated in *P. salmonis* broth (PSB) or on its solid equivalent PSA at 18°C (Henriquez et al., 2016). *E. coli* strains were propagated in Luria-Bertani broth (LB, Difco) or on tryptic soy agar plates (TSA, Difco) at 37°C. Plasmids were introduced into *E. coli* strains by transformation and into *P. salmonis* strains through mating with the donor strain *E. coli* S17-1*λpir* (Simon et al., 1983). Where necessary, antibiotics (Sigma) were used at the following concentrations: kanamycin at 50 μg/ml and polymyxin B at 10 μg/ml. Any potentially infectious residue was inactivated by autoclaving before disposal according to the biosafety protocols of ADL Diagnostic Chile. Plasmids pCR2.1 TOPO (Invitrogen) and pJQK (Quandt and Hynes, 1993) were manipulated according to standard cloning procedures.

**Table 1:** Bacterial strains and plasmids used in this study.

ID	Description	Source/reference
PM15972A1	Sequenced EM-90-like *P. salmonis* field isolate	Bohle et al., 2014
PM15972	Animalized challenge *P. salmonis* PM15972A1 strain	This study
Δ*wzx*	*P. salmonis* PM15972 in-frame deletion mutant with *wzx* gene deletion (Δ33-443*)	This study
Δ*wcaJ*	*P. salmonis* PM15972 in-frame deletion mutant with *wcaJ* gene deletion (Δ31-413)	This study
TOP10	*E. coli* cloning strain	Invitrogen
S17-1λ*pir*	*E. coli m*ating strain with plasmid RP4 inserted into the chromosome. Supports the replication of R6K plasmids	Simon et al., 1983
pCR2.1-TOPO	Cloning plasmid. Kanamycin and ampicillin resistances	Invitrogen
pJQK	Mobilizable plasmid with RP4 origin of transfer. Kanamycin resistance	Quandt & Hynes, 1993
pCR*wzx*	pCR2.1-TOPO derivative containing a 812-bp fragment resulting from the amplification with *wzx*_F1 and R4 primers	This study
pCR*wcaJ*	pCR2.1-TOPO derivative containing a 669-bp fragment resulting from the amplification with *wcaJ*_F1 and R4 primers	This study
pJQ*wzx*	pJQK derivative containing the *Bam*HI-*Xba*I fragment of pCR*wzx*	This study
pJQ*wcaJ*	pJQK derivative containing the *Bam*HI-*Xba*I fragment of pCR*wcaJ*	This study

*Deletion comprising amino acid range according to the coding sequence annotated in the accession n° CP012413.

### Sequence Analysis

The *P. salmonis* PM15972A1 genome sequence was retrieved from the GenBank database (accession n° CP012413). The annotation was examined with CLC Genomics Workbench software (Qiagen), looking for genes encoding functions related to the biosynthesis of precursors of LPS and EPS. Sequence alignments were accomplished with BLAST. For comparative purposes, sequences from identified gene clusters were compared with those annotated in the KEGG database (https://www.genome.jp/kegg).

### Construction of In-Frame Deletion Mutant Strains

All primers used in this study are listed in **Table 2**. The sequences of the *wzx* and *wcaJ* genes of *P. salmonis* PM15972A1 were obtained from the NCBI (National Center for Biotechnology Information, http://www.ncbi.nlm.nih.gov/) (Bohle et al., 2014). *P. salmonis wzx* or *wcaJ* in-frame deletion mutants were engineered by overlapping PCR following a protocol previously described for *Brucella* (Conde-Alvarez et al., 2006). Briefly, two DNA fragments obtained with the primer pairs *wzx*_F1/*wzx*_R2 and *wzx*_F3/*wzx*_R4 were ligated by PCR extension, and the resulting fragment was cloned into pCR2.1-TOPO supported by the TOP10 *E. coli* strain (**Figure S1**). Subcloning into the pJQK plasmid was achieved by restriction digestion of the corresponding pCR2.1 TOPO derivative and transformation into the S17-1λ*pir E. coli* donor strain. The resulting pJQK derivative was introduced into *P. salmonis* by conjugation. Transconjugants carrying the pJQ*wzx* plasmid integrated into the chromosome by a single crossover event were then selected on PSA containing kanamycin and polymyxin B. Allelic exchange between the chromosomal gene and the mutagenized plasmid copy was achieved by a second crossover event and was counter-selected on PSA containing 5% sucrose (**Figure S2**). An identical procedure but different primers were used for the construction of the pJQ*wcaJ* mutator plasmid and the corresponding *P. salmonis* Δ*wcaJ* mutant strain. The presence of the respective mutated genes in each strain was confirmed by the analysis of PCR amplicons generated with primers F1-R4. Mutated genes were also sequenced by Sanger chemistry to confirm the interchange with the wild-type allele in the resulting mutant clones. Bacterial strains were stored in glycerol stocks at -70°C.

**Table 2:** Sequences of oligonucleotides used throughout this study.

Name	Sequence (5’-3’)	T_m_ (°C)	Reference
*wzx*_F1	ACGTGCATTGGGGGATAAAT	54.7	This study
*wzx*_R2	CACCCCAAACTCGTGCTAAT	55.3	This study
*wzx*_F3	ATTAGCACGAGTTTGGGGTGTGGCTGCAATTCGGAAAAA	67.3	This study
*wzx*_R4	ATAAATGCTGCTTGCGACCT	55.1	This study
*wcaJ*_F1	TTGTTGAGCCGCTTGAATGG	56.3	This study
*wcaJ*_R2	ATCCACTGCTAGCGACCAAC	57.3	This study
*wcaJ*_F3	GTTGGTCGCTAGCAGTGGATGATTACAGGGTGGGCACAAG	68.6	This study
*wcaJ*_R4	AACAATCAACACGCCATTGA	53.1	This study
*ifnγ*_F	CCGTACACCGATTGAGGACT	56.5	Valenzuela et al., 2013
*ifnγ* _R	GCGGCATTACTCCATCCTAA	54.6	Valenzuela et al., 2013
*il1β*_F	CCCCATTGAGACTAAAGCCA	54.6	Valenzuela et al., 2013
*il1β*_R	GCAACCTCCTCTAGGTGCAG	57.6	Valenzuela et al., 2013
*tnfα*_F	AGGCTTTTTCCCAGGGC	55.7	Valenzuela et al., 2013
*tnfα*_R	GAGTCCGAATAGCGCCAA	54.9	Valenzuela et al., 2013
*elf1α*_F	GCCCCTCCAGGAYGTYTACAA	57.2-61.0	Sepulveda et al., 2013
*elf1α* _R	CCACACGGCCCACRGGTAC	61.0-63.7	Sepulveda et al., 2013
*rpoD_*F	GCTCCGACAAGCAACATCGGC	61.5	Vera, 2013
*rpoD_*R	GCGCTCTGCCGCTTCCTCAA	63.1	Vera, 2013
2958_F	GAGTATGGCGGTAACGAGCA	57.0	This study
2958_R	GACTGATACGGCAAGCGTCT	57.2	This study
2966_F	CGTCCGATTATTCAATTTGCTCCTG	56.3	This study
2966_R	TCACTCCATGTATGAGCAATAGGG	56.2	This study
SRS-F	GCTGTGCCCAGAACTTTAG	53.6	This study
SRS-R	GACCACTRCCTTTACCAAAC	53.3	This study

### Growth Kinetics, Bacterial Counts, and Self-Agglutination Tests

100 µL aliquots of deep-frozen *P. salmonis* wild-type and mutant strains were streaked onto PSA plates and incubated for 2 days at 18°C. By this time, a homogeneous bacterial lawn had formed and we proceeded to suspend a loopful of bacteria in 1 mL of buffered saline solution (BSS: 8.5 g/L NaCl, 2.0 g/L K_2_HPO_4_, 2.0 g/L KH_2_PO_4_, pH 6.4), transferred them onto new PSA plates, and incubated as mentioned above. Bacterial suspensions (OD_600_ = 1.0) were prepared by mixing bacteria with 5 mL of cold BSS in a conical tube kept at 10°C. For growth kinetics, 96-well microplates were inoculated with 150 µL/well of 1:100 bacterial suspensions in PSB and incubated during 70 h. Hourly reads were realized with an EPOCH2 spectrophotometer (Biotek, USA). By contrast, self-agglutination tests lasted 8 h, but used bacterial suspensions prepared in PSB that were held in plastic cuvettes at 18°C and pH 7.2. Reads were collected each hour with a densitometer (Ultrospec 10, GE LifeSciences). In order to determine bacterial counts, aliquots of log_10_ dilutions in PSB were seeded on PSA plates in triplicates and incubated at 18°C for one week. Acriflavine tests were carried out using fresh bacterial suspension in BSS mixed with identical volumes (10 µL) of 0.1% acriflavine on a glass slide.

### Bactericidal Action of *Salmo salar* Naïve Serum

The complement killing activity of naïve serum was evaluated following a previously described protocol (Saavedra et al., 2017). Atlantic salmon (*Salmo salar*) smolts with an average body weight of 96 g were caudally punctured, and naïve serum was obtained after blood clotting for 2 h at 4°C and centrifugation at 2,000 × g for 5 min. Samples from 5 fish were pooled. Bacterial inoculum was set at ~10^7^ colony forming units (cfu)/mL and was treated with undiluted fish serum for 3 h at 18°C. Heat-inactivated (44°C, 20 min) serum aliquots were used as negative controls.

### LPS Purification and Analysis

LPS was extracted from *P. salmonis* wild-type and mutants following a previously described method (Yi and Hackett, 2000). 15 mL of a fresh culture with OD_600_ = 1.9 were harvested, which corresponded to roughly 10 mg of freeze-dried bacteria. LPS was isolated with a commercial RNA isolation reagent (Trizol) and subsequently resuspended in 200 µL of PBS. Discontinuous SDS-PAGE electrophoresis was run on 15% and 20% polyacrylamide stacking/solving gels. Gels were silver-stained according to a well-known protocol (Hitchcock and Brown, 1983). Alternatively, LPS was extracted without organic solvent following a protocol described before (Renzi et al., 2016). Briefly, 1 mL of bacterial suspension in PBS, adjusted to OD_600_ = 1.0, was pelleted by centrifugation and subsequently lysed with Laemmli buffer. Samples were boiled and treated twice with proteinase K (50 µg/mL final protease concentration, overnight at 37°C). Crude protein samples and LPS prepared following the recommendations by Renzi and colleagues were used for independent Western blot assays. These samples were eletrophoresed in 15% polyacrylamide gels. Nitrocellulose membranes were blotted according to a conventional protocol, using semi-dry transfer equipment (Bio-Rad) for 35 min at 20 V. These blots were incubated with polyclonal serum extracted from a rabbit that had been immunized with heat-inactivated *P. salmonis* PM15972A1 cells. Signals were then revealed with an HRP-conjugated secondary antibody and a suitable chemical substrate.

### Biofilm Formation Assessment

Biofilm formation assays were performed following a previously described method (Pal et al., 2019), with few modifications. Briefly, the wells of a sterile 24-well polystyrene microtiter plate were inoculated with 1 mL of *P. salmonis* wild-type and mutant strain suspensions adjusted in PSB to obtain ~7.0 × 10^6^ cfu/well. Sterile PBS was used as a negative control. Plates were incubated at 18°C for 30 days under static conditions. Then, wells were washed twice with BSS to remove the planktonic cells and stained with 0.1% crystal violet (CV) for 15 min. Excess CV was removed by renewed washing with BSS, and bound dye was solubilized in 1 mL of 33% acetic acid. Solubilized CV was subsequently transferred to a new microplate and its absorbance was spectrophotometrically determined at 620 nm. This same measurement was done on negative-control wells containing bacteria-free medium in order to account for non-specific and abiotic factors. Total biofilm formation was calculated by subtracting the average OD_620_ value of the stained control wells from the OD_620_ value of the stained biofilm (Naves et al., 2008). Calculated values were finally normalized to those obtained for the *P. salmonis* PM15972A1 positive control.

### RNA Purification and Gene Expression Analysis

So as to evaluate possible differences in gene expression triggered by the treatment with *P. salmonis* wild-type and mutant strains, RNA extraction and cDNA synthesis were performed as described by Mancilla et al. (2018). RNA was purified using the EZNA Total RNA Kit I (Omega Bio-Tek, Norcross, GA, USA). After DNAse I digestion, purified RNA was stored at -70°C. Reverse transcription was performed using the M-MLV RT Kit (Promega, USA) and synthesized cDNA was stored at -20°C. Gene expression was analyzed by qPCR using the Maxima SYBR^®^ Green qPCR Master Mix (Thermo Scientific) in a StepOne PCR machine (Applied Biosystems, Waltham, MA, USA), whereby each reaction was performed using 250 nM of primers (**Table 2**) and 1.0 µL of 1:2 diluted cDNA as a template. The thermal profile included an initial step of 10 min at 95°C, followed by 40 cycles of 15 s at 95°C and 1 min at 60°C. The fold change of gene expression was calculated according to the 2^-ΔΔCt^ method (Pfaffl, 2001), using the expression of the elongation factor 1α (*efl1*α) as a normalizer and the expression of all markers under control conditions as a calibrator.

### Cell Infection Assays

SHK-1 cells (passage 72) were cultured without antibiotics in Leibovitz’s L-15 medium supplemented with 10% fetal bovine serum (FBS) at 20°C in 75-cm^2^ flasks. Cells were seeded into 24-well plates at a concentration of ~1.0 × 10^5^ cell/well, incubated for 24 h at 20°C and then infected with the selected *P. salmonis* strain at a multiplicity of infection (MOI) = 10. Purified nucleic acid samples were collected from the cell culture at 5 and 10 days post infection (dpi). Cell damage induced by *P. salmonis* in SHK-1 cell monolayers was determined by the release of the cytosolic enzyme lactate dehydrogenase (LDH) into the medium using a commercial Cytotoxicity LDH Detection Kit (Thermo Scientific). LDH activity levels were analyzed in 50 µL aliquots of cell-free supernatant obtained from each well. Additionally, the supernatant of cells lysed with L-15 medium containing 1% Triton X-100 was used as a total lysis control (maximum LDH liberation) and the supernatant of uninfected cells was used as a negative control (basal LDH liberation). Optical densities measured at 490 nm and 680 provided the basis for calculations as recommended by the manufacturer of the detection kit.

The *in vitro* infectivity of *P. salmonis* was assessed using an endpoint dilution assay (TCID_50_) as described early, with few modifications (House et al., 1999). CHSE-214 cells were cultured in 96-well flat-bottom microplates in antibiotic-free L-15 Medium supplemented with 10% FBS and incubated at 20°C until they reached confluence (~5.0 × 10^4^ cells/well). As inoculum, bacterial suspensions (~5.0 × 10^7^ cfu/mL) were prepared in L-15 supplemented with 2% FBS, and log_10_ dilutions were made up to the 10^th^ dilution. Monolayers were infected (8 wells/dilution), incubated for 15 days at 18°C, and then visually inspected for the presence of cytopathic effects. TCID_50_ were calculated according to the Spearman–Kärber algorithm (Hierholzer and Killington, 1996).

### *In Vivo* Evaluation

Atlantic salmon smolts were confirmed as “pathogen free” using RT-PCR routine diagnostic methods for IPNV, ISAV, PRV, *P. salmonis* and *Renibacterium salmoninarum*. After their transfer to the experimental center, fish were tagged using Passive Integrated Transponder tags (PIT tags). The animals were allowed to acclimatize to seawater for 21 days and fed *ad libitum*; they were maintained at 14 ± 1°C, with a water change rate of 1.2 - 1.5/hour, pH of 7.0 - 8.0, a photoperiod of 24 light hours, and a specific feeding rate of 1.5 - 2%. Prior to any handling and marking, fish were anesthetized with benzocaine. Finally, euthanasia was performed using an overdose of anesthetic. All efforts were made to provide best growth conditions and minimize suffering.

The *in vivo* challenge was carried out on 420 fish with an approximate weight of 90 - 100 g. These fish were uniformly allocated to three 0.5 m^3^ tanks, yielding initial densities of 25 - 30 kg/m³ and 140 fish/tank. 35 fish of each tank comprised a single treatment group that received 0.1 mL of bacterial suspension (either *P. salmonis* wild-type, *P. salmonis* Δ*wzx*, or *P. salmonis* Δ*wcaJ*) or vehicle (BSS). Fresh bacterial suspensions (OD_600_ = 0.7) were used to prepare the inocula that were administered to the fish *via* intraperitoneal injection. In parallel, a sample of each inoculum was utilized to perform bacterial counts at the time of injection.

Fish were monitored daily for 30 days, and mortalities were removed from the tanks. As for tanks 1 and 2, mortalities were recorded and subjected to necropsy. Additionally, PCR of internal organ samples were done to assess for the presence of *P. salmonis*. The third tank was intervened on days 5, 12 and 20 post infection, removing three live fish/group to collect head kidney tissue samples for gene expression analysis.

### Statistical Analysis

Data obtained from the bactericidal serum effect studies were tested for normal distribution using the Shapiro–Wilk test and were subsequently analyzed with the non-parametric Mann–Whitney rank-sum test. For biofilm quantification and cytotoxicity assays, the mean and standard deviation values were compared with the Kruskal-Wallis test and Dunn’s multiple comparison test as a *post-hoc* analysis (p < 0.05). These same tests were applied to the mean and standard deviation values of the fold change of gene expression, which are given in terms of relative expression. PCR efficiencies were determined by linear regression analysis of sample data using LinReg-PCR. With regard to *in vivo* testing, the percentages of cumulative mortalities were analyzed using the Kaplan-Meier method, and the differences were evaluated using the log-rank test performed with GraphPad Prism v6.0.

## Results

### Identification of Genes Encoding Functions Related to Polysaccharide Biosynthesis

**Figure 1:**
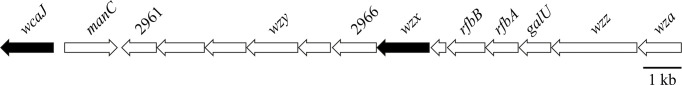
P*. salmonis* PM15972 LPS gene cluster. Genetic organization of the ~18 kb cluster containing genes putatively involved in the biosynthesis of LPS and EPS. Mutagenesis gene targets *wzx* and *wcaJ* are marked in black. For a detailed description of gene functions, see **Table 3**.

BLAST searches and annotation analyses allowed for the identification of a ~18 kb cluster carrying 15 genes putatively involved in the biosynthesis of LPS (**Figure 1**). Those genes presumably encode enzymes involved in furnishing (glycosyltransferases), O-chain transport across the membrane, and precursor biosynthesis (**Table 3**). An O-antigen flippase (*wzx*, KW89_2967, 462 amino acids), which catalyzes the transport of the O-antigen unit across the inner membrane, was predicted. We also found genes encoding proteins possibly participating in the polymerization of the O-antigen (*wzy*, KW89_2964; *wzz*, KW89_2972). Surprisingly, homologs of the *waaL* O-antigen ligase, which facilitates the transference of polymerized O-antigen to the core-lipid A, could not be identified. A putative undecaprenyl-phosphate glucose phosphotransferase or *wcaJ* (KW89_2959, 466 amino acids) was found to be located in that same cluster, but upstream of *wzx* and in close proximity to another gene encoding an S-layer protein. The enzyme encoded by *wcaJ* is allegedly responsible for the initiation of the synthesis of colanic acid, which is part of the EPS and the capsule of the *Enterobacteriaceae* family members. In detail, undecaprenyl-phosphate glucose phosphotransferase accounts for the transfer of glucose-1-phosphate onto the carrier lipid undecaprenyl phosphate. Interestingly, a second cluster of ~8.5 kb containing genes for the biosynthesis of a capsular polysaccharide was found (**Figure S3** and **Table S1**). Some genes of this cluster show similarities with orthologs in *Francisella philomiragia* and *F. noatunensis* subsp. *orientalis*.

**Table 3:** Description of the gene products from the LPS biosynthesis gene cluster.

ORF	Gene	Length*	Predicted function
KW89_2959	*wcaJ*	466	Undecaprenyl-phosphate glucose phosphotransferase
KW89_2960	*manC*	465	Mannose-1-phosphate guanylyltransferase/mannose-6-phosphate isomerase
KW89_2961	–	308	NAD-dependent epimerase/dehydratase family protein
KW89_2962	–	418	Glycosyltransferase family 4 protein
KW89_2963	–	363	Glycosyltransferase family 4 protein
KW89_2964	*wzy*	452	Oligosaccharide repeat unit polymerase
KW89_2965	–	284	Glycosyltransferase family 2 protein
KW89_2966	–	390	Poly(glycerophosphate) glycerophosphotransferase
KW89_2967	*wzx*	462	Oligosaccharide flippase family protein
KW89_2968	–	135	Hypothetical protein
KW89_2969	*rfbB*	334	dTDP-glucose 4,6-dehydratase
KW89_2970	*rfbA*	290	Glucose-1-phosphate thymidylyltransferase
KW89_2971	*galU*	282	UTP-glucose-1-phosphate uridylyltransferase
KW89_2972	*wzz*	751	Polysaccharide biosynthesis tyrosine autokinase
KW89_2973	*wza*	381	Polysaccharide biosynthesis/export family protein

*Length of coding sequence in amino acid.

According to the literature, a knock-out mutation of *wzx* or *wcaJ* would carry a detrimental effect on the biosynthesis of LPS and EPS, respectively. Therefore, altered virulence phenotypes of the corresponding mutants could be expected if those structures play a role in the pathogenesis of piscirickettsiosis.

### Mutagenesis of *wzx* and *wcaJ* Genes

**Figure 2:**
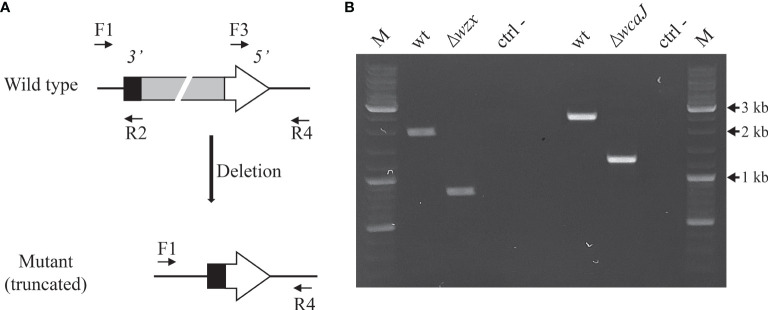
Genotypic characterization of Δ*wzx* and Δ*wcaJ P. salmonis* mutant strains. **(A)** Position of primer pairs to accomplish PCR overlap and expected engineered mutant allele. **(B)** PCR analysis of wild-type and mutant strains using flanking primers F1 and R4 for each gene. ctrl-, non-template control. M,10 kb DNA ladder.

Since the target genes are likely to be part of operon units, the mutagenesis approach was engineered to yield in-frame deletion mutants. Mutator plasmids were designed using the pJQK genetic tool, a suicide plasmid already tested in *P. salmonis* (Mancilla et al., 2018). We have chosen a strategy similar to that used in other bacterial models, replacing the wild-type gene by a short, afunctional copy of the same gene. This way, we could avoid polar effects and remove antibiotic resistance markers (**Figure 2A** and **Figure S4**). The interchanged alleles remaining after the mutagenesis process were detected and confirmed by conventional PCR (**Figure 2B**). It shall be mentioned that the mutant strains have lost their kanamycin resistance markers and displayed minimal inhibitory concentration (MIC) values at control, wild-type levels.

### *P. salmonis* Mutants Display Particular *In Vitro* Phenotypes

**Figure 3:**
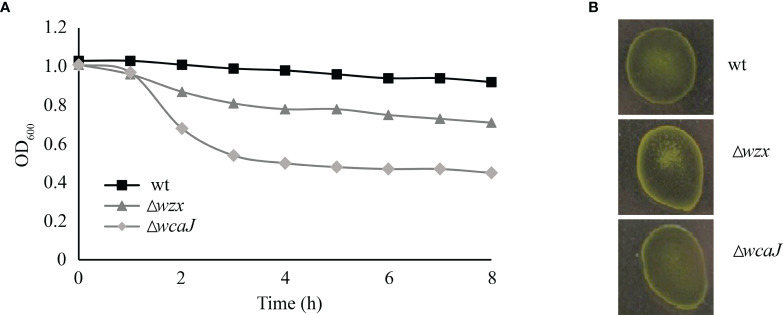
Self-agglutination assessment in *P. salmonis* strains. **(A)** Kinetic OD measurement of wild-type and mutant bacterial suspensions. **(B)** Acriflavine slide test.

Cell envelope mutants frequently present colony phenotypes that are easily distinguished on agar plates. However, isolated colonies from the *P. salmonis* mutants described in this study looked macroscopically identical to the wild-type strain (not shown). Growth kinetics in liquid medium showed no differences between the mutants and the parent strain (**Figure S5**). Considering prior reports on the self-agglutination ability of mutants with cell envelope defects, we studied our *P. salmonis* strains in this regard. On the one hand, we measured the optical density of bacterial suspensions in liquid medium, and we conducted acriflavine tests. In liquid medium, mutant strains displayed a higher self-agglutination capacity than their parent strain, and this characteristic was particularly pronounced in Δ*wcaJ* (**Figure 3A**). On the contrary, the acriflavine test was positive only for the Δ*wzx* strain, where cell clumps became evident (**Figure 3B**). Since LPS is the main target of some antibiotics, such as polymyxin B, we assessed the mutant strains’ MIC to this antibiotic. Unexpectedly, similar MIC values were determined for the parent and mutant phenotypes (8 - 16 µg/mL).

**Figure 4:**
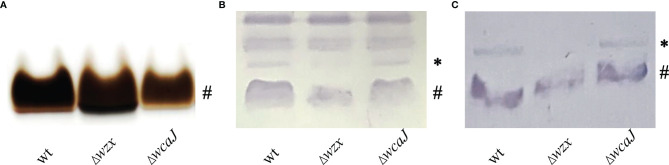
LPS characterization. **(A)** Silver-stained LPS fractions from each strain extracted with organic solvent. **(B)** Western blots of crude protein samples and **(C)** LPS extracted without organic solvent. Blots were incubated with rabbit polyclonal serum against *P. salmonis* wild-type strain. (#) refers to core-lipid A species, while (*) indicates the core-lipid A plus O-antigen, which is absent in Δ*wzx* mutant.

In a Δ*wzx* genetic background, it is expected to find O-chain-deficient LPS species with a lower molecular mass when compared with the parent strain. That feature can be explored by electrophoretic profiling, so we examined the profiles of LPS samples from the parent and mutant strains in a 15% conventional acrylamide gel. Consistent with previous reports (Kuzyk et al., 1996; Vadovic et al., 2007), we visualized a single-band profile with an apparent molecular mass of 11 kDa, but did not find any differences among tested strains (not shown). Upon increasing the resolution of polyacrylamide gels, we observed subtly augmented electrophoretic mobility for the Δ*wzx* LPS fraction, when compared with the parent and Δ*wcaJ* mutant strains (**Figure 4A**). This pattern resembled the canonical core-lipid A profile. In a next step, we used crude protein samples that would be expected to carry LPS, performed Western blots and finally incubated with immunoreactive rabbit serum against *P. salmonis* wild-type. While samples corresponding to the parent and Δ*wcaJ* mutant strains yielded similar profiles, the Δ*wzx* mutant lacked a band (**Figure 4B**). An identical pattern was observed on a Western blot of LPS samples extracted without organic solvent and extensively treated with proteinase K (**Figure 4C**).

**Figure 5:**
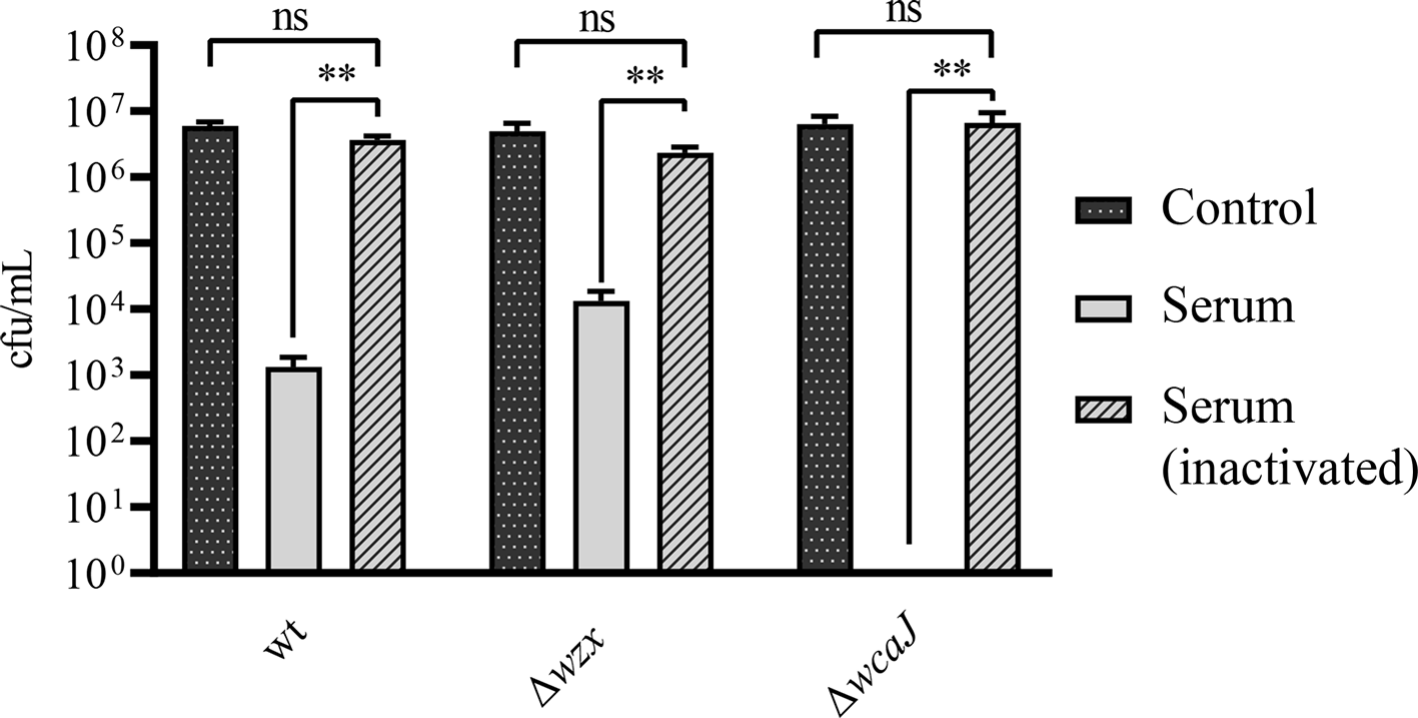
Bactericidal action of naïve serum. Survival is denoted as colony counts recovered after serum treatment. Control corresponds to bacterial suspension without treatment. Differences were statistically significant (**p < 0.01). No significant differences are denoted by "ns"

Some bacterial pathogens are able to resist the killing activity of naïve serum. While this applies to *P. salmonis*, the resistance of this pathogen seems to depend on its genetic background (Saavedra et al., 2017). In this regard, we found the parent and the Δ*wzx* mutant strains to display partial susceptibility to salmon naïve serum. The Δ*wcaJ* strain, however, proved to be completely susceptible to the serum treatment and was unable to survive (**Figure 5**).

**Figure 6:**
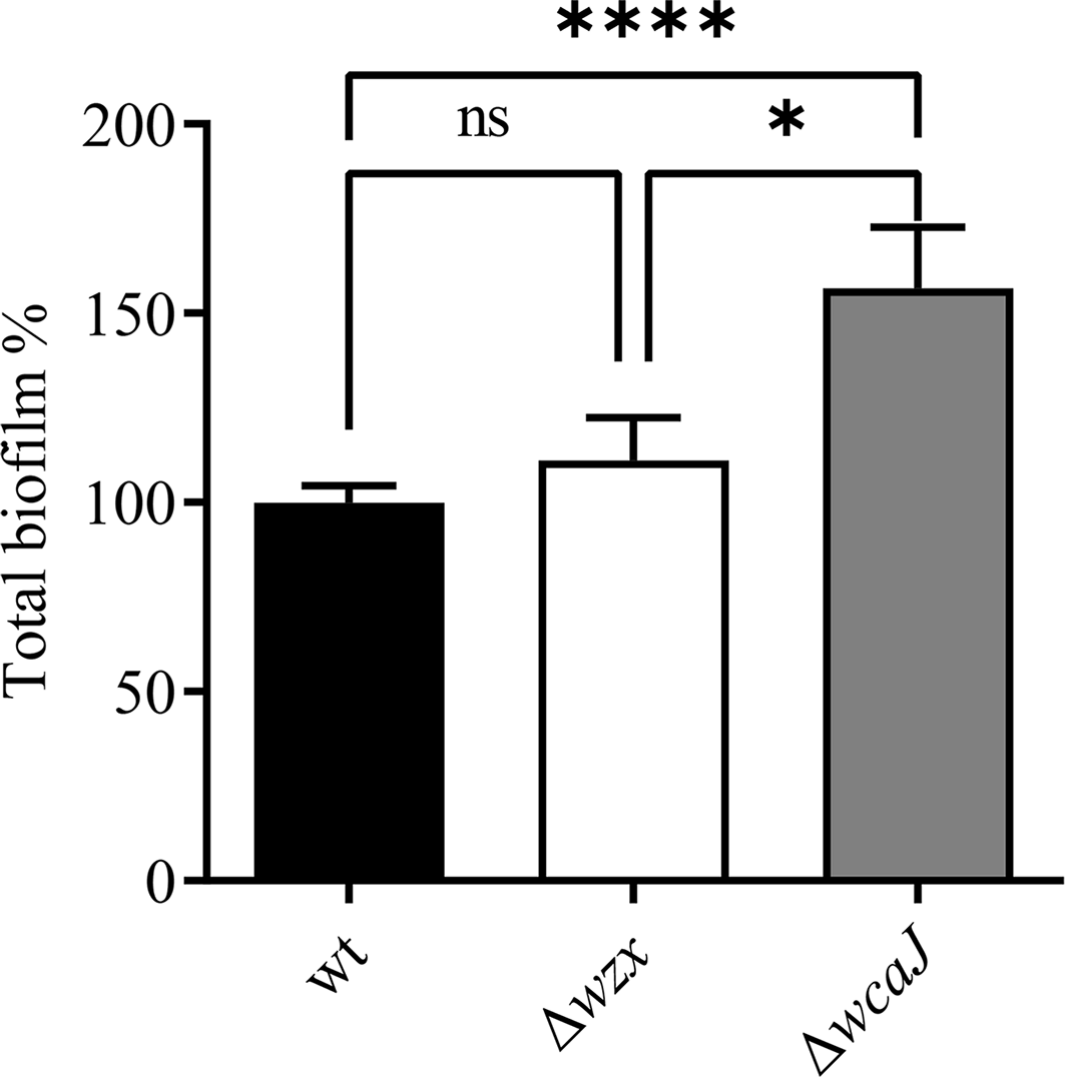
Biofilm formation assay. Ability of biofilm formation expressed as percentage of crystal violet staining using the wild-type strain as reference. Enhanced ability of biofilm production by the Δ*wcaJ* strain was statistically significant (*p < 0.05 and ****p < 0.001). No significant differences (ns) were found between Δ*wzx* and its parent strain.

Available experimental evidence does not unequivocally confirm the role of the *wcaJ* gene in the biosynthesis of EPS. While a knock-out mutation of this gene in the fish pathogen *Edwarsiella tarda* caused an impairment of biofilm production (Yu et al., 2015), enhanced biofilm formation was described for *Klebsiella pneumoniae* carrying the same mutation (Pal et al., 2019). As for our *P. salmonis* strains, the evaluation of biofilm production after 30 days of incubation showed that the disruption of *wcaJ* is associated with enhanced biofilm formation. The corresponding mutant strain formed up to 50% more biofilm than the wild-type strain. In contrast, no differences were observed between the Δ*wzx* and wild-type strains (**Figure 6**).

### Mutants Are Distinguishable in LDH Cytotoxicity Assays

**Figure 7:**
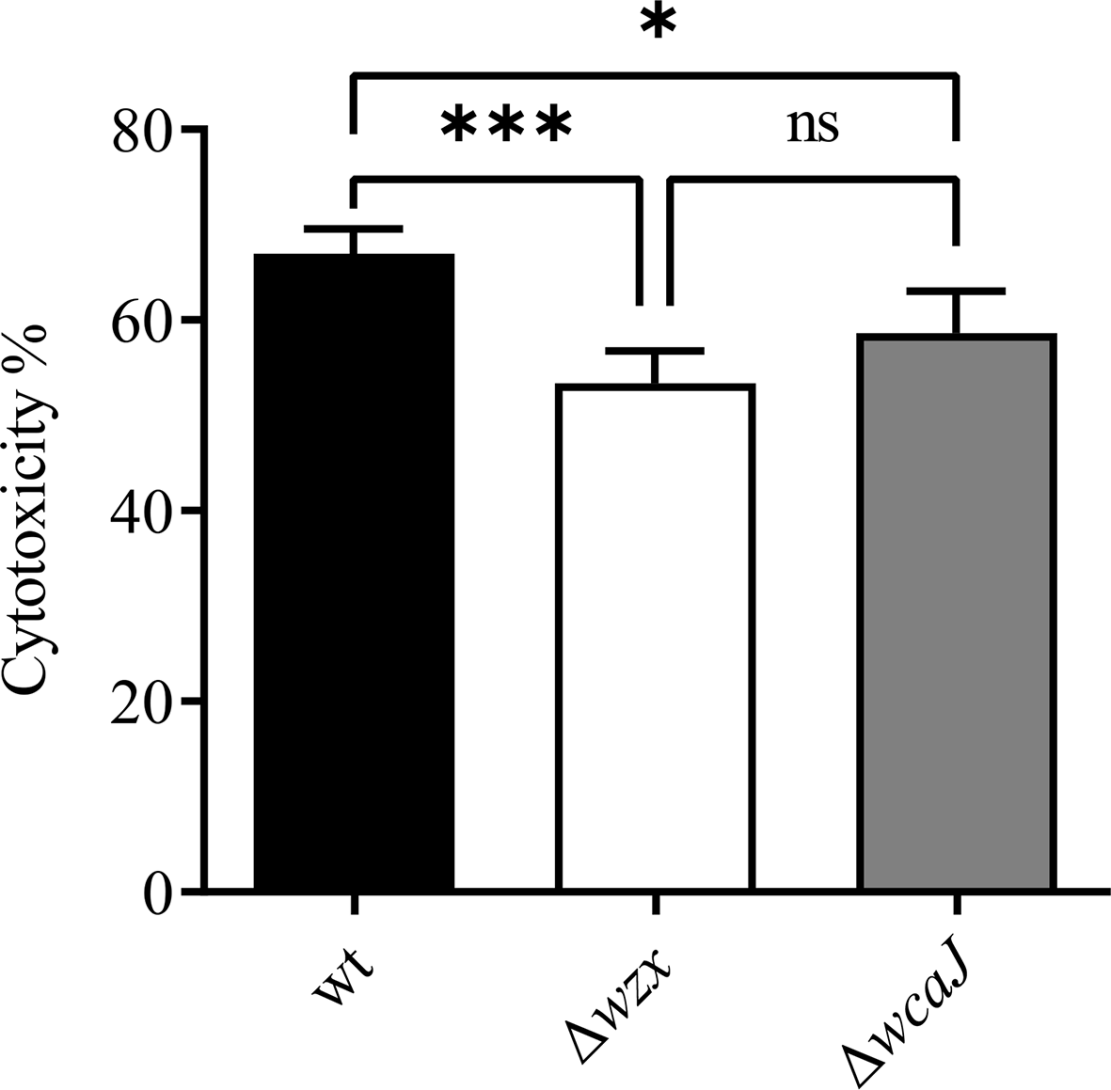
Cytotoxicity assay. LDH release by SHK-1 cells expressed as percentage of enzymatic activity using the wild-type as reference. Differences were statistically significant (*p < 0.05 and ***p < 0.005). No significant differences are denoted by "ns".

The CHSE-214 cell line has been described as susceptible to infection with *P. salmonis*. Hence, we hypothesized that using comparable amounts of bacteria to perform TCID_50_ studies would reveal potential differences in the virulence of mutant strains. We thus conducted the assays at a similar MOI. Notwithstanding, titers reached by the mutants and the parent strain were in the same order of magnitude and ranged from 6.0 - 6.7 × 10^5^ TCID_50_/ml. In a second, independent experiment, calculated titers were somewhat higher, but still largely identical across *P. salmonis* strains, ranging from 8.0 - 9.3 × 10^5^ TCID_50_/ml. In order to consolidate these findings, we decided to further assess the virulence of *P. salmonis* mutants in SHK-1 cells. The direct observation of cytopathic effects is less reliable in SHK-1 cells, which is why we opted for the quantification of LDH release and activity as an alternative, more sensitive measure of the *in vitro* virulence of *P. salmonis* strains in this cell line. To this end, monolayers of SHK-1 cells were infected at a lower MOI and incubated at 18°C for 10 days. In this model, both mutants exhibited lowered cytotoxicity when compared with their parent strain (**Figure 7**). The Δ*wzx* mutant was the most attenuated one.

### Mutants Trigger a Differential Proinflammatory Response *In Vitro* and *In Vivo*

**Figure 8:**
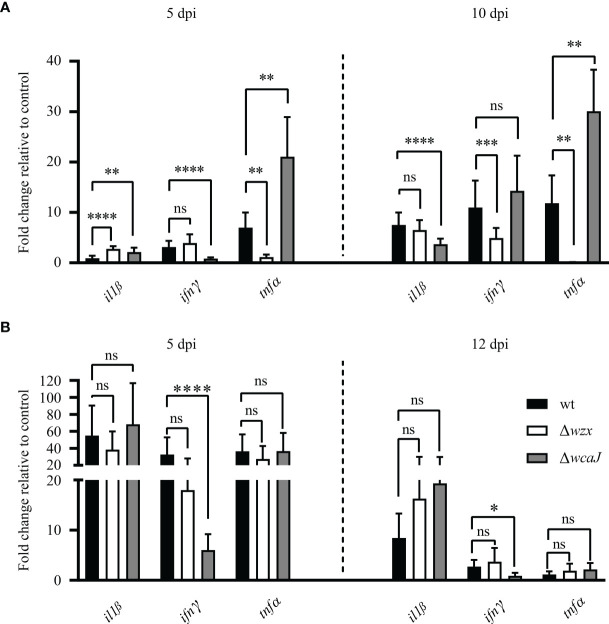
Proinflammatory marker gene expression after bacterial infection. Relative gene expression of cytokine genes *il1ß, ifnγ* and *tnfα* as observed in the **(A)** *in vitro* SHK-1 cell model of *P. salmonis* infection and **(B)** in intraperitoneally challenged Atlantic salmon smolts. Differences were statistically significant (*p < 0.05, **p < 0.01, ***p < 0.005 and ****p < 0.001). No significant differences are denoted by "ns".

Pathogen mutants with cell envelope defects tend to induce differential patterns of gene expression of immune response markers in host cells. We tested this hypothesis for *P. salmonis* strains by determining the expression profiles of a proinflammatory cytokine gene panel in SHK-1 cells, sampled at 5 or 10 dpi. All strains were able to stimulate a proinflammatory response, but clear differences became evident (**Figure 8A**). The Δ*wzx* strain upregulated the expression of *il1β* and downregulated the expression of *tnfα* at 5 dpi. At 10 dpi, *tnfα* and *ifnγ* were downregulated. Interestingly, cells infected with the Δ*wcaJ* strain showed a notable upregulation of *tnfα* at both points in time (>20-fold change), while *il1β* was found to be upregulated at 5 dpi but dropped below reference values at 10 dpi. As for the expression of *ifnγ* after infection with the Δ*wcaJ* strain, a clear downregulation was registered at 5 dpi. Gene expression was also examined in samples derived from the *in vivo* assay. An upregulation of the expression of *il1β* and *tnfα* was recorded at 5 dpi in fish infected with all strains tested (**Figure 8B**). Consistent with the pattern observed *in vitro*, a downregulation of *ifnγ* expression was observed at 5 dpi in the group infected with the Δ*wcaJ* strain. Interestingly, at 12 dpi, expression profiles of all markers tended to level off at reference levels, with the exception of the *ifnγ* transcript. The expression pattern for this cytokine gene was characterized by persistent downregulation in fish treated with the Δ*wcaJ* strain. Extensive mortality was registered in groups infected with the parent and Δ*wcaJ* strains; only fish from the Δ*wzx*-challenged group survived up to 20 dpi. Therefore, no comparison of gene expression could be made at this sampling point in time. We highlight the reliability of our qPCR data, which it can be deduced from the normalizer behavior across samples (**Figure S6**).

### Δ*wzx* Mutant Presents Lower Virulence in *S. salar*

**Figure 9:**
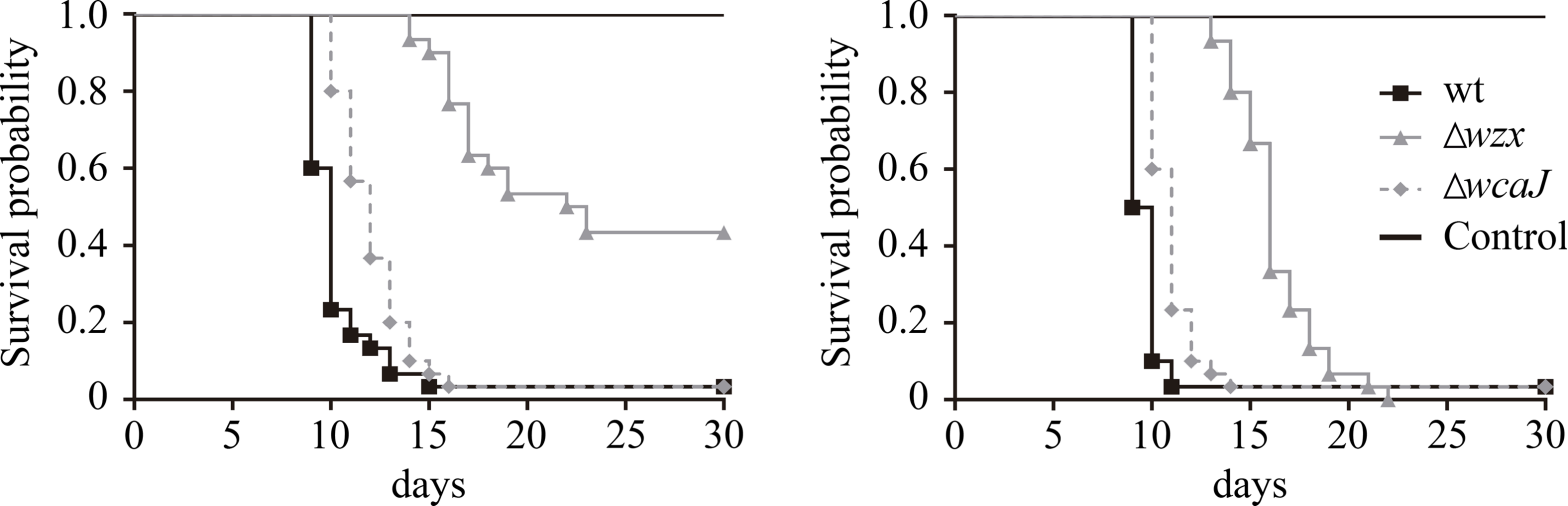
*In vivo* virulence of the mutants and wild-type strain. Results obtained from two tanks, each containing three treatment and one control group each consisting of 35 fish. Differences in the probability of survival were statistically significant for the Δ*wzx* mutant group in both tanks (*p* < 0.001).

The *in vivo* assay was carried out infecting *S. salar* smolt cohorts with similar amounts of *P. salmonis* strains *via* intraperitoneal injection. According to our experience with the challenge strain, the horizontal transmission of *P. salmonis* PM15972 is negligible in the period set for the experiment, and any mortality occurring within that time frame may be interpreted as an effect of the injected material. The target concentration of the inoculum was set at 1.0 × 10^7^ cfu/fish (Mancilla et al., 2018). Precisely, individual fish were inoculated with 3.0 × 10^7^ and 2.6 × 10^7^ cfu of the parent strain and Δ*wzx* mutant, respectively, while the Δ*wcaJ* treatment group received 1.3 × 10^6^ cfu/fish. As shown in **Figure 9**, the group injected with the parent strain accumulated almost 100% mortality within a few days. Treatment with the Δ*wcaJ* mutant induced a similar mortality curve, although it was slightly displaced in time. Fish infected with *P. salmonis* Δ*wzx* had a significantly higher probability of survival in both tanks. Necropsies conducted on dead fish revealed pathognomonic signs of piscirickettsiosis in internal organs, regardless of the challenge strain (not shown).

## Discussion

Classical works done on the subject of colony variants were reported at the very beginning of microbiology. These studies did not only provide insight into a biological phenomenon, but also laid the foundation for the development of bacterial attenuated vaccines (Van der Woude and Baumler, 2004). Notwithstanding, colony variants are hard to be observed for *P. salmonis.* In the present study, we had to use a variety of indirect techniques to establish the differences between the engineered strains.

Our findings are consistent with response evasion mechanisms conferred by LPS and EPS for *P. salmonis*. On the one hand, LPS with large, heteropolymeric O-antigens displays a typical ladder-like banding pattern of the repeating sugar units in SDS-PAGE, but this pattern cannot be reproduced with *P. salmonis* LPS (**Figure 4A**). In addition, the Δ*wzx* mutant presented levels of resistance to serum and MIC values very similar to its parent strain. In light of the alteration of LPS, this was an unexpected finding, but similar results have been reported for the Gram-negative fish pathogen *Aliivibrio salmonicida*. The LPS structure of that bacterium resembles a lipooligosaccharide (LOS) rather than a classical, long O-chain furnished LPS, and a structural LPS mutant did not present alterations of the serum resistance phenotype when compared with its parent strain (Norstebo et al., 2018). So as to explain the reduced susceptibility to polymyxin B manifested by *P. salmonis*, we may refer to findings obtained in naturally virulent, rough species of the *Brucella* genus. These pathogens have a truncated O-chain LPS and are not only resistant to the action of complement, but also to that of positively charged antimicrobial peptides, including polymyxin B (Stranahan and Arenas-Gamboa, 2021). Taking into account these precedents, our results obtained in serum survival and polymyxin B MIC tests may be related to *P. salmonis* having evolved carrying an LPS with an atypical short O-antigen, suggesting that the LOS structure conferred an evolutionary advantage to this pathogen.

**Figure 10:**
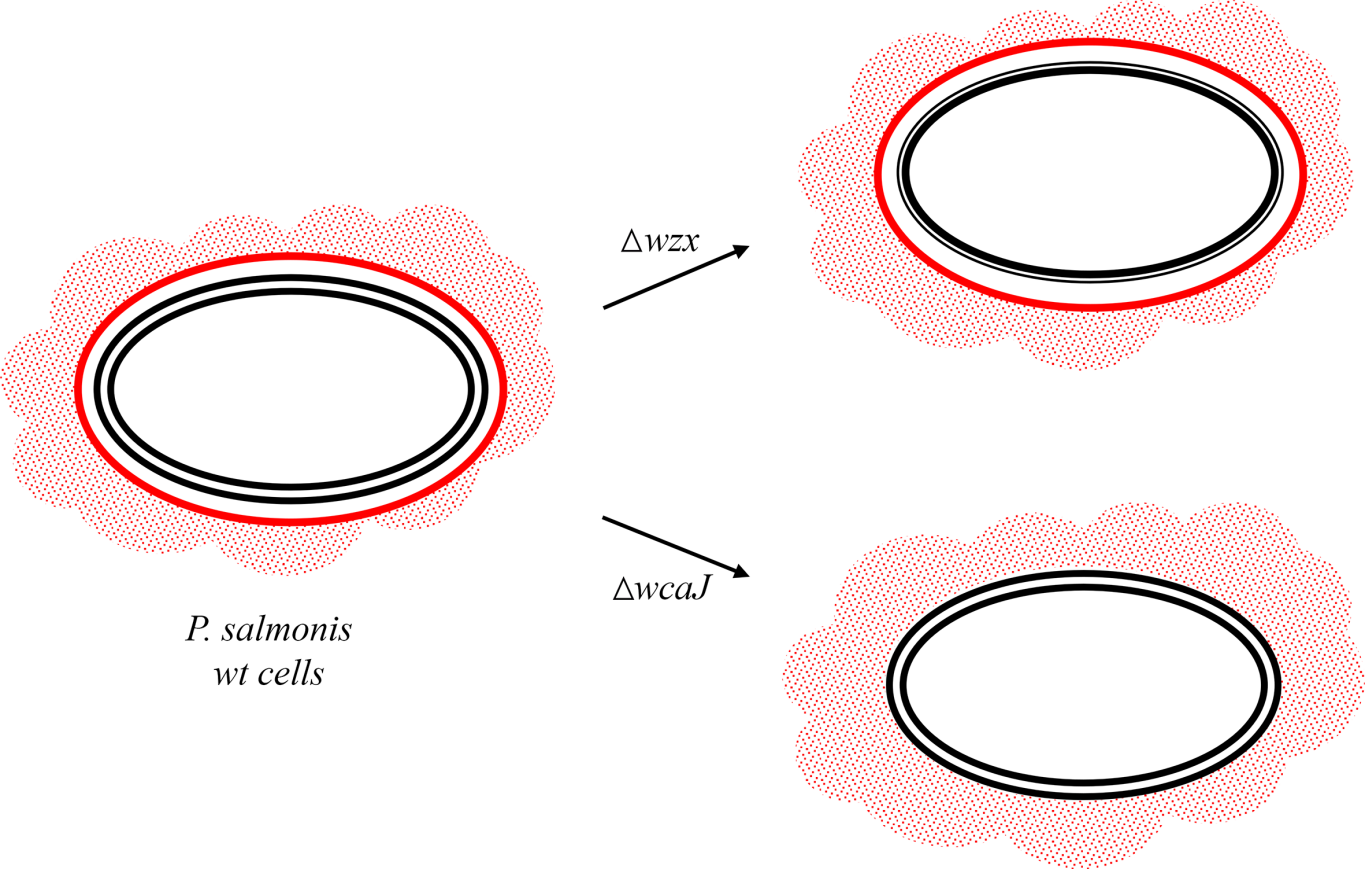
Model of cell envelope defects in *P. salmonis* mutants. In the Δ*wzx* mutant, the most external leaflet of the lipid bilayer (black, double line) is devoid of O-chain LPS. In contrast, Δ*wcaJ* behaves as a capsule-deficient strain (single red line) that retains its ability to form biofilm (red dotted area).

On the other hand, the Δ*wcaJ* mutant proved to be fully susceptible to serum, and this phenotype closely resembles capsule-deficient strains that have already been described in the literature (Renzi et al., 2016; Tan et al., 2020). We may hypothesize that the loosely attached EPS of the Δ*wcaJ* mutant results in a decreased ability to sterically hinder the access of complement components to the bacterial surface, thereby increasing its susceptibility to serum (Rautemaa and Meri, 1999; Holland and Lambris, 2002). In this context, it is tempting to speculate that the EPS is masking some binding sites of complement proteins, which may remain exposed in the Δ*wcaJ* mutant. This idea also supports the notion that a fraction of EPS is attached to the membrane. Beyond this speculation, it is clear that the biosynthesis of the *P. salmonis* LPS is not affected by the disruption of *wcaJ*, which is probably participating in a biosynthesis pathway of a different glycan. The differential effects of *wzx* and *wcaJ* mutations on the cell envelope structure of *P. salmonis* are illustrated in **Figure 10**.

Even though our study did not aim at resolving the structure of *P. salmonis* LPS, our findings allow to draw some conclusions on this topic. According to the work of Vinogradov and colleagues, the oligosaccharide backbone structure of *P. salmonis* LPS is consistent with a LOS structure. We observed a single band pattern with no higher molecular mass bands in silver-stained gels, but samples obtained from the Δ*wzx* mutant under mild conditions were lacking an immunoreactive band characteristic of the wild-type strain (**Figure 4B**). Our interpretation of this band is that it does represent a carbohydrate antigen, most probably the core-lipid A plus O-antigen. The fact that this band is absent in silver-stained gels may be explained by the labile nature of the covalent bond linking this carbohydrate unit to the core-lipid A. This hypothesis is strongly supported by Western blot patterns of LPS samples extracted without organic solvent (**Figure 4C**). Additional evidence comes from Δ*wzx* mutant LPS samples purified by the Westphal phenol method, which exhibit only the band corresponding to the O-antigen-devoid core-lipid A structure (**Figure S7**). Increased sensitivity to organic solvents of the bond bridging the O-chain and core-lipid A has also been observed in unrelated bacteria such as *Pseudomonas aeruginosa* (Abeyrathne et al., 2005). Therefore, our results confirm the findings of Vinogradov et al. (2013) and extend their conclusion to a putative single O-antigen unit bound to the core-lipid A backbone of wild-type *P. salmonis* LPS.

It has been reported that *P. salmonis* hexacyl lipid A reminds canonical *Enterobacteriaceae* structure (Vadovic et al., 2007). Furthermore, it has been demonstrated for members of this family that colanic acid forms part of the EPS and disruption of *wcaJ* (also known as *wecA*) hampers the biosynthesis of this molecule, thereby affecting biofilm formation and other phenotypic traits (Raetz and Whitfield, 2002). Indeed, biofilm formation can be diversely affected by *wcaJ* deletion and thus we deduced that *wcaJ* may be involved in the biosynthesis of EPS in *P. salmonis*. Contrary to our expectations, the Δ*wcaJ* mutant yielded more biofilm than the parent strain. The mechanisms underlying this phenotype are not clear and findings in the literature are contradictory, e.g., a *K. pneumoniae* Δ*wcaJ* mutant was shown to produce relatively higher amounts of biofilm (Pal et al., 2019). One possibility is that *P. salmonis* does not synthesize colanic acid, but a compositionally different EPS as part of a capsule. Since undecaprenyl phosphate, also known as bactoprenol, is a common substrate for the biosynthesis of diverse bacterial glycans, the disruption of *wcaJ* may affect the availability of this precursor in the EPS pathway. Thus, material may be left for export and contribute to the unorganized biofilm architecture rather than being available for the assembly of a capsule covering the bacterium. In support of this notion, a capsular polysaccharide biosynthesis gene cluster was evidenced by sequence similarity. This finding argues in favor of a capsular antigen in *P. salmonis*, but also provides genetic evidence for the modifications related to diacetylated pseudaminic acid residues found in the LPS core structure described previously (Vinogradov et al., 2013).

Altogether, we provide evidence supporting the hypothesis that the *P. salmonis* LPS O-chain is transported across the membrane *via* a Wzx-Wzy dependent pathway and that its disruption results in a structural LPS mutant. Moreover, complete LPS is required for full virulence of the pathogen in the intraperitoneal model of infection in *S. salar* as it can be deduced from the results of our *in vivo* assay. Further studies are needed to elucidate the genetic basis of oligosaccharide core modifications, which is decorated with chemical groups that may help to evade innate immune mechanisms such as antimicrobial peptides and complement. In the case of EPS, future research is warranted since our results suggest that this biopolymer may be more complexly structured than assumed to date and that the presence of a capsule cannot be discarded. At this point in time, however, we also cannot rule out a role of EPS in pathogenicity. In light of this seemingly contradictory conclusion, we note the fact that our intraperitoneal infection model does not necessarily mimic the natural route of transmission of piscirickettsiosis. Additionally, the EPS seems to be masking some pathogen-associated molecular pattern (PAMP) that modulates the immune response in the host, as can be inferred from the differential gene expression patterns prompted by the Δ*wcaJ* mutant. Indeed, our results suggest that the hypothetical exposed PAMP in that mutant downregulates the expression of *ifnγ*, *in vitro* and *in vivo*, and upregulates *tnfα in vitro*, while the Δ*wzx* mutant does not seem to modify the expression profiles of cytokine genes studied *in vivo*, but yields a significant downregulation of *tnfα in vitro.* With regard to the cause of differences between *in vitro* and *in vivo* results, it must be considered that the *in vivo* response reflects transcript levels, which are representative only for the tissue tested. Measured values may not be directly comparable to those detected in samples derived from a stimulated cell line. Further research is needed to give a satisfactory explanation for the differences detected in both models, but the response induced by the mutants clearly accounts for an altered host-pathogen interaction as compared with the one elicited by the wild-type strain.

In sum, our findings on *P. salmonis* cell envelope components are consistent with a stealth strategy, in which the pathogen evades the detection by the host immune system in the early stages of infection. This view is also complementary to the imbalance towards a suppressive immune response that takes place during the late intracellular replication of *P. salmonis* cells.

## Data Availability Statement

The original contributions presented in the study are included in the article/**Supplementary Material**. Further inquiries can be directed to the corresponding authors.

## Ethics Statement

The animal study was reviewed and approved by ADL Diagnostic Chile ethics committee.

## Author Contributions

VH carried out the *in vitro* characterization of bacterial strains, along with NO. Gene expression analysis was also performed by VH in collaboration with YY. JS, and OA performed the mutagenesis experiments. PB provided approval for publication. MM conceived and designed the study with the help of OA, and both wrote the paper. All authors contributed to the article and approved the submitted version.

## Funding

This research was funded by Corporación de Fomento de la Producción de Chile, CORFO, grants 16ITE1-71014 and 17PIDE-80700, and Fondo de Desarrollo Científico y Tecnológico, FONDECYT/ANID, postdoctoral grant 3170340. The authors declare that this study received funding from Blue Genomics SpA and ADL Diagnostic Chile. The funders were not involved in the study design, collection, analysis, interpretation of data, the writing of this article or the decision to submit it for publication.

## Conflict of Interest

Authors VH and OA were employed by Blue Genomics SpA.

The remaining authors declare that the research was conducted in the absence of any commercial or financial relationships that could be construed as a potential conflict of interest.

## Publisher’s Note

All claims expressed in this article are solely those of the authors and do not necessarily represent those of their affiliated organizations, or those of the publisher, the editors and the reviewers. Any product that may be evaluated in this article, or claim that may be made by its manufacturer, is not guaranteed or endorsed by the publisher.
