# Supplementary material for: Complete Lipopolysaccharide of Piscirickettsia salmonis Is Required for Full Virulence in the Intraperitoneally Challenged Atlantic Salmon, Salmo salar, Model
Source: Front Cell Infect Microbiol. 2022 Mar 18;12:845661. doi: 10.3389/fcimb.2022.845661 (PMC8972169; doi:10.3389/fcimb.2022.845661)
Supplement: Supplementary file 1 [file Presentation_1.pptx]

## Slide 1
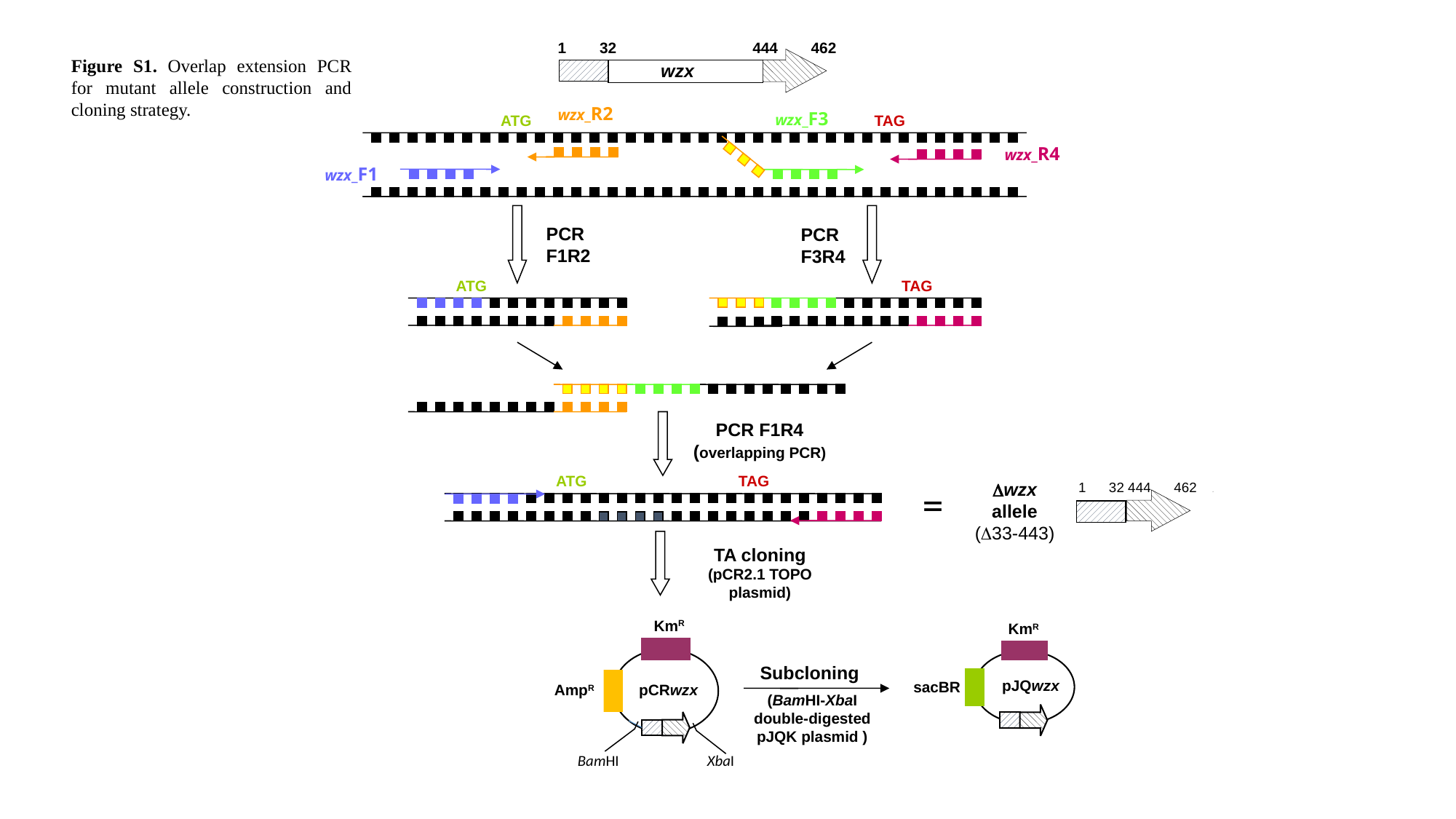

1 32
444 462
Figure S1. Overlap extension PCR for mutant allele construction and cloning strategy.
wzx
wzx_R2
wzx_F3
ATG
TAG
wzx_R4
wzx_F1
PCR F1R2
PCR F3R4
ATG
TAG
PCR F1R4 (overlapping PCR)
ATG
TAG
=
Δwzx allele (D33-443)
 1 32 444 462
TA cloning (pCR2.1 TOPO plasmid)
 KmR
AmpR
 KmR
Subcloning
(BamHI-XbaI double-digested pJQK plasmid )
pJQwzx
sacBR
pCRwzx
XbaI
BamHI

## Slide 2
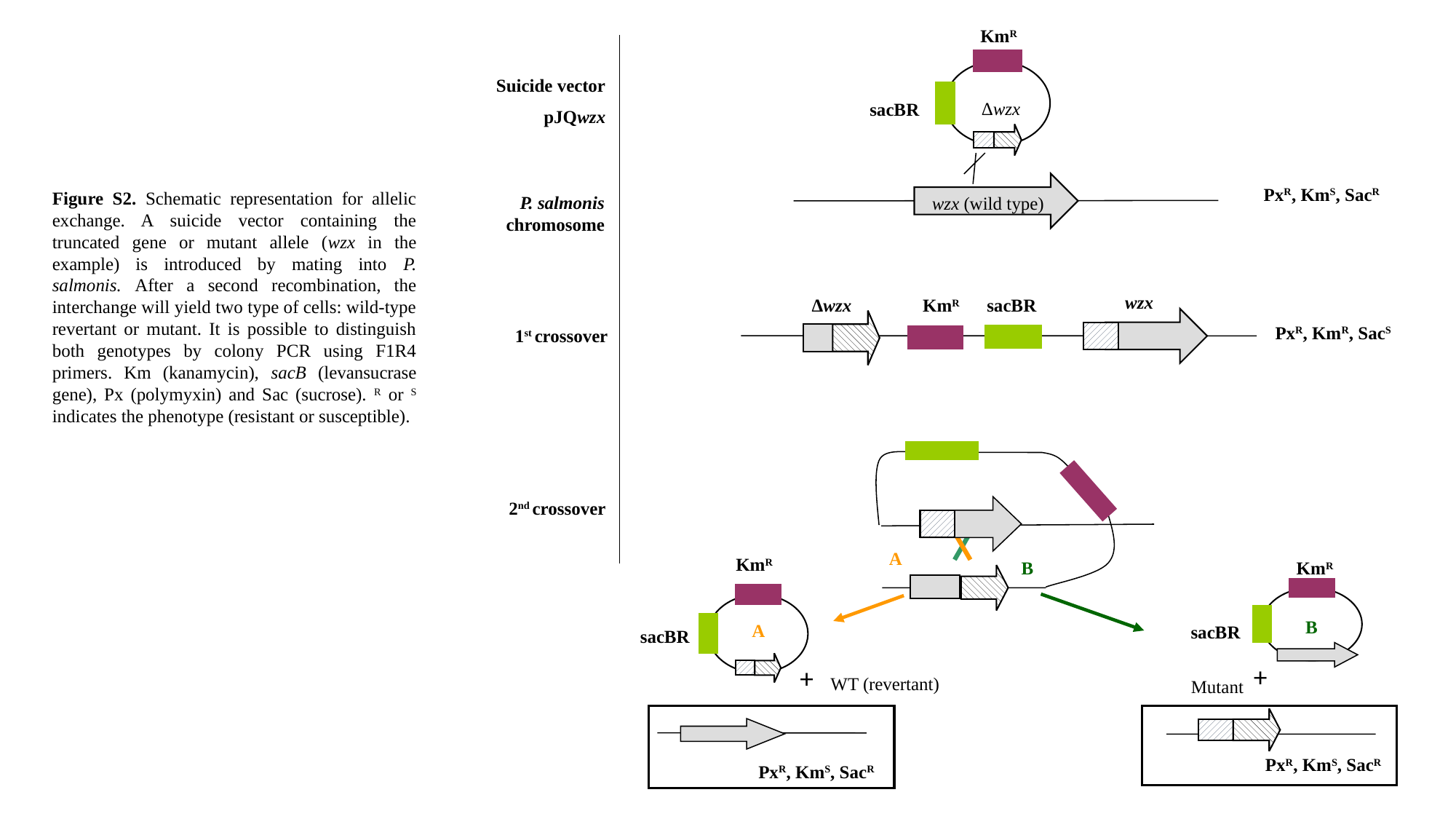

KmR
sacBR
Suicide vector
 pJQwzx
Δwzx
PxR, KmS, SacR
Figure S2. Schematic representation for allelic exchange. A suicide vector containing the truncated gene or mutant allele (wzx in the example) is introduced by mating into P. salmonis. After a second recombination, the interchange will yield two type of cells: wild-type revertant or mutant. It is possible to distinguish both genotypes by colony PCR using F1R4 primers. Km (kanamycin), sacB (levansucrase gene), Px (polymyxin) and Sac (sucrose). R or S indicates the phenotype (resistant or susceptible).
P. salmonis chromosome
wzx (wild type)
wzx
Δwzx
 KmR sacBR
PxR, KmR, SacS
1st crossover
A
B
2nd crossover
 KmR
sacBR
 KmR
sacBR
B
A
+
+
WT (revertant)
Mutant
PxR, KmS, SacR
PxR, KmS, SacR

## Slide 3
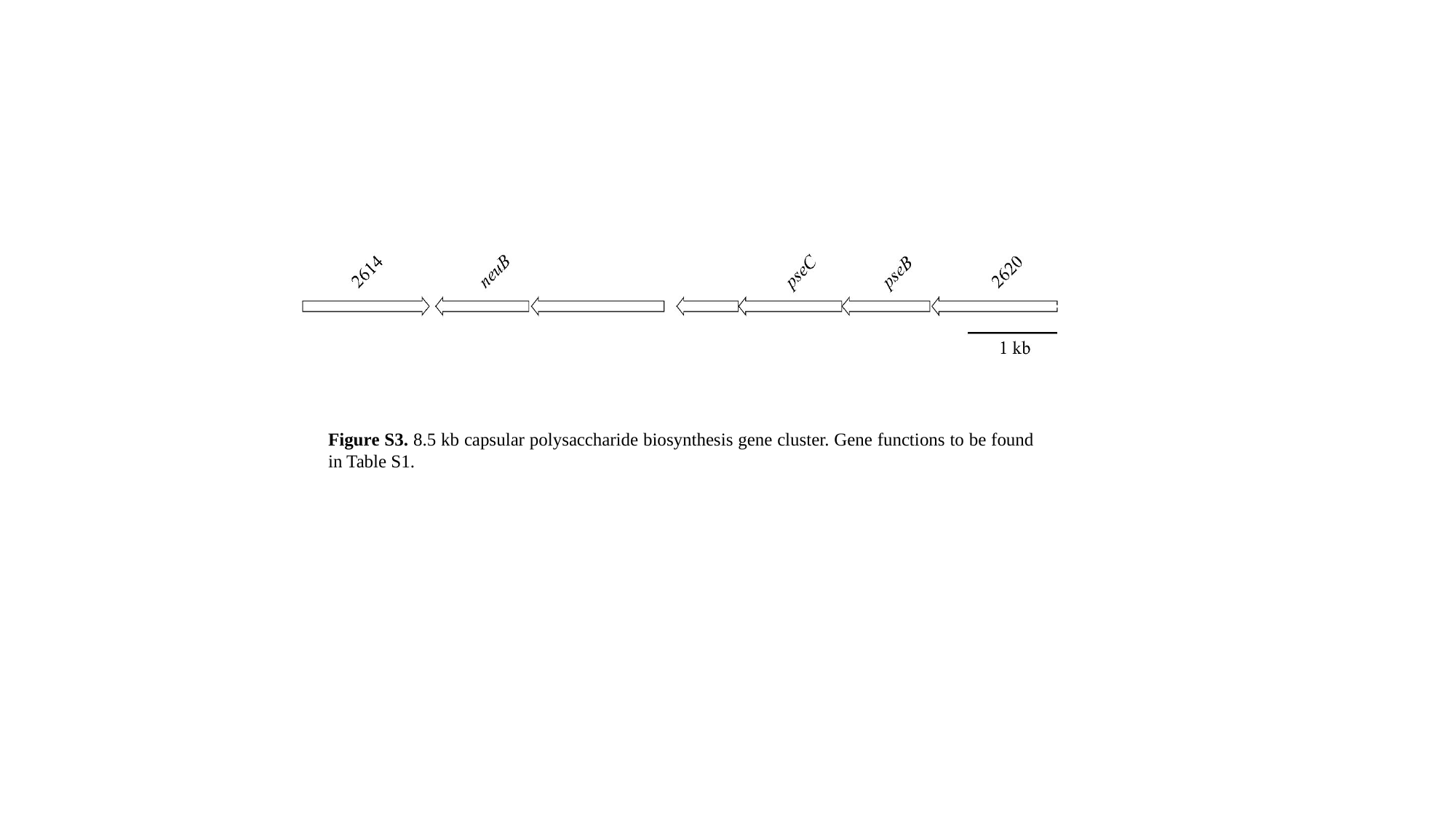

Figure S3. 8.5 kb capsular polysaccharide biosynthesis gene cluster. Gene functions to be found in Table S1.

## Slide 4
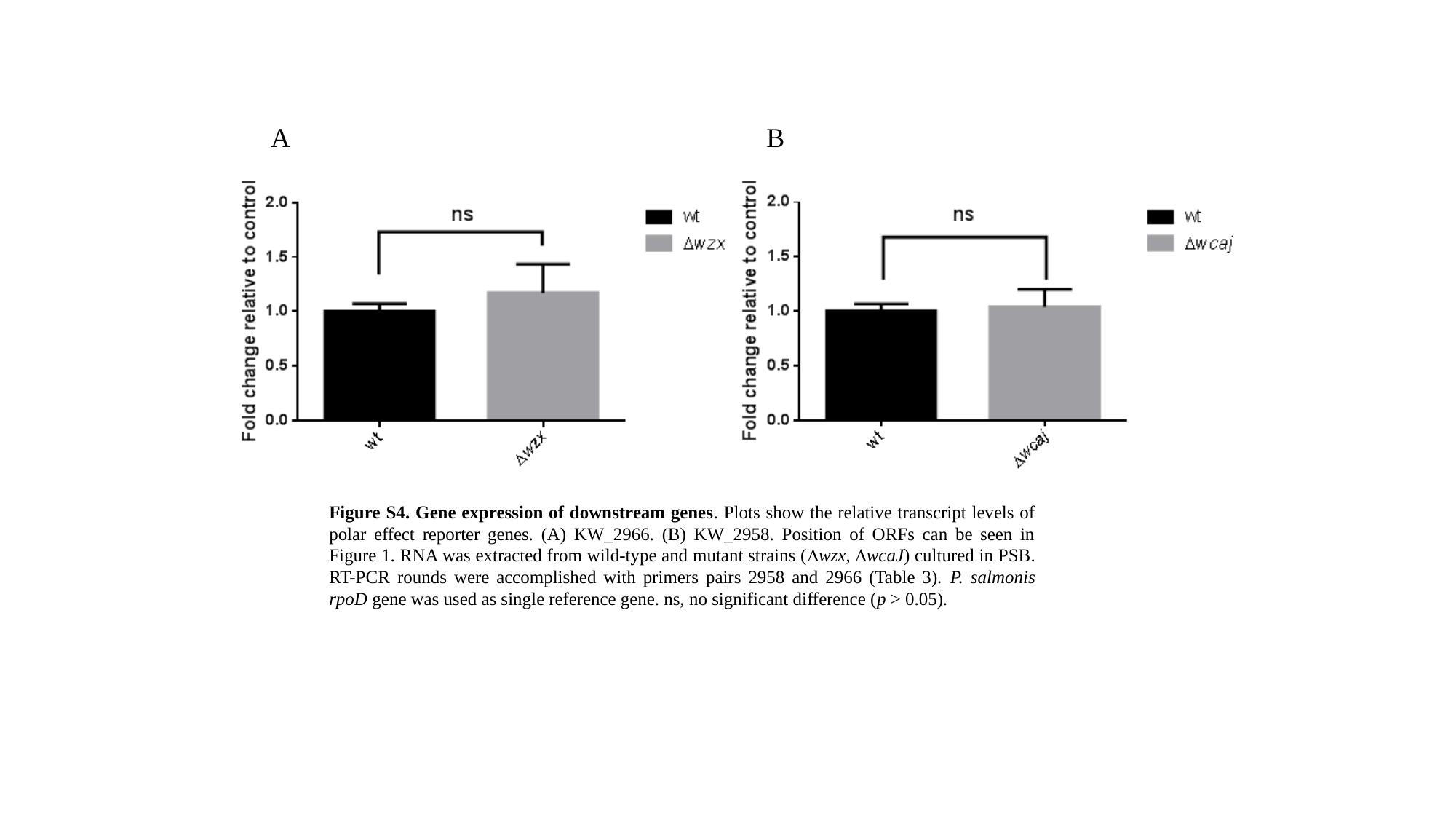

A
B
Figure S4. Gene expression of downstream genes. Plots show the relative transcript levels of polar effect reporter genes. (A) KW_2966. (B) KW_2958. Position of ORFs can be seen in Figure 1. RNA was extracted from wild-type and mutant strains (Δwzx, ΔwcaJ) cultured in PSB. RT-PCR rounds were accomplished with primers pairs 2958 and 2966 (Table 3). P. salmonis rpoD gene was used as single reference gene. ns, no significant difference (p > 0.05).

## Slide 5
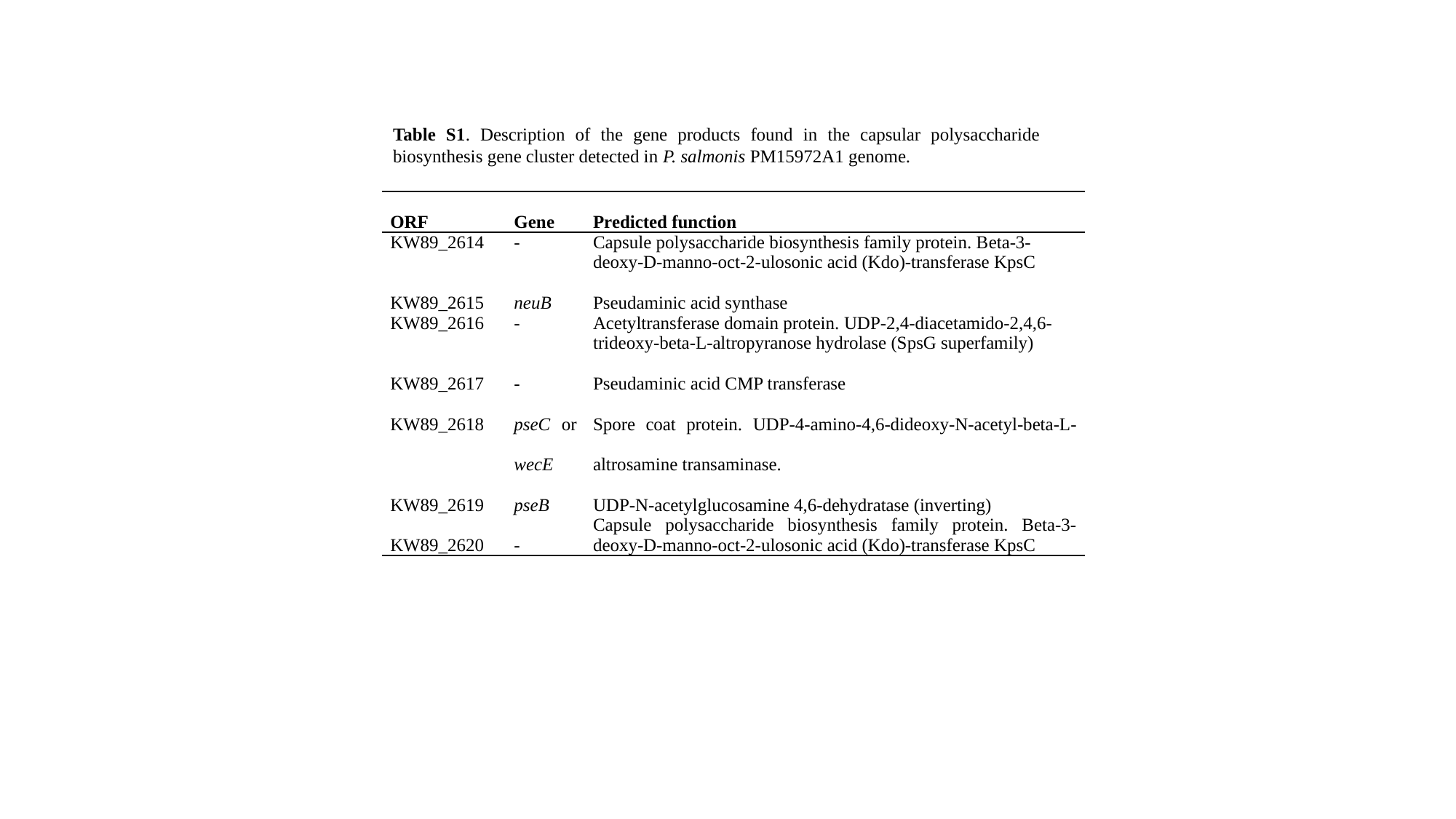

Table S1. Description of the gene products found in the capsular polysaccharide biosynthesis gene cluster detected in P. salmonis PM15972A1 genome.
| ORF | Gene | Predicted function |
| --- | --- | --- |
| KW89\_2614 | - | Capsule polysaccharide biosynthesis family protein. Beta-3-deoxy-D-manno-oct-2-ulosonic acid (Kdo)-transferase KpsC |
| KW89\_2615 | neuB | Pseudaminic acid synthase |
| KW89\_2616 | - | Acetyltransferase domain protein. UDP-2,4-diacetamido-2,4,6-trideoxy-beta-L-altropyranose hydrolase (SpsG superfamily) |
| KW89\_2617 | - | Pseudaminic acid CMP transferase |
| KW89\_2618 | pseC or wecE | Spore coat protein. UDP-4-amino-4,6-dideoxy-N-acetyl-beta-L-altrosamine transaminase. |
| KW89\_2619 | pseB | UDP-N-acetylglucosamine 4,6-dehydratase (inverting) |
| KW89\_2620 | - | Capsule polysaccharide biosynthesis family protein. Beta-3-deoxy-D-manno-oct-2-ulosonic acid (Kdo)-transferase KpsC |

## Slide 6
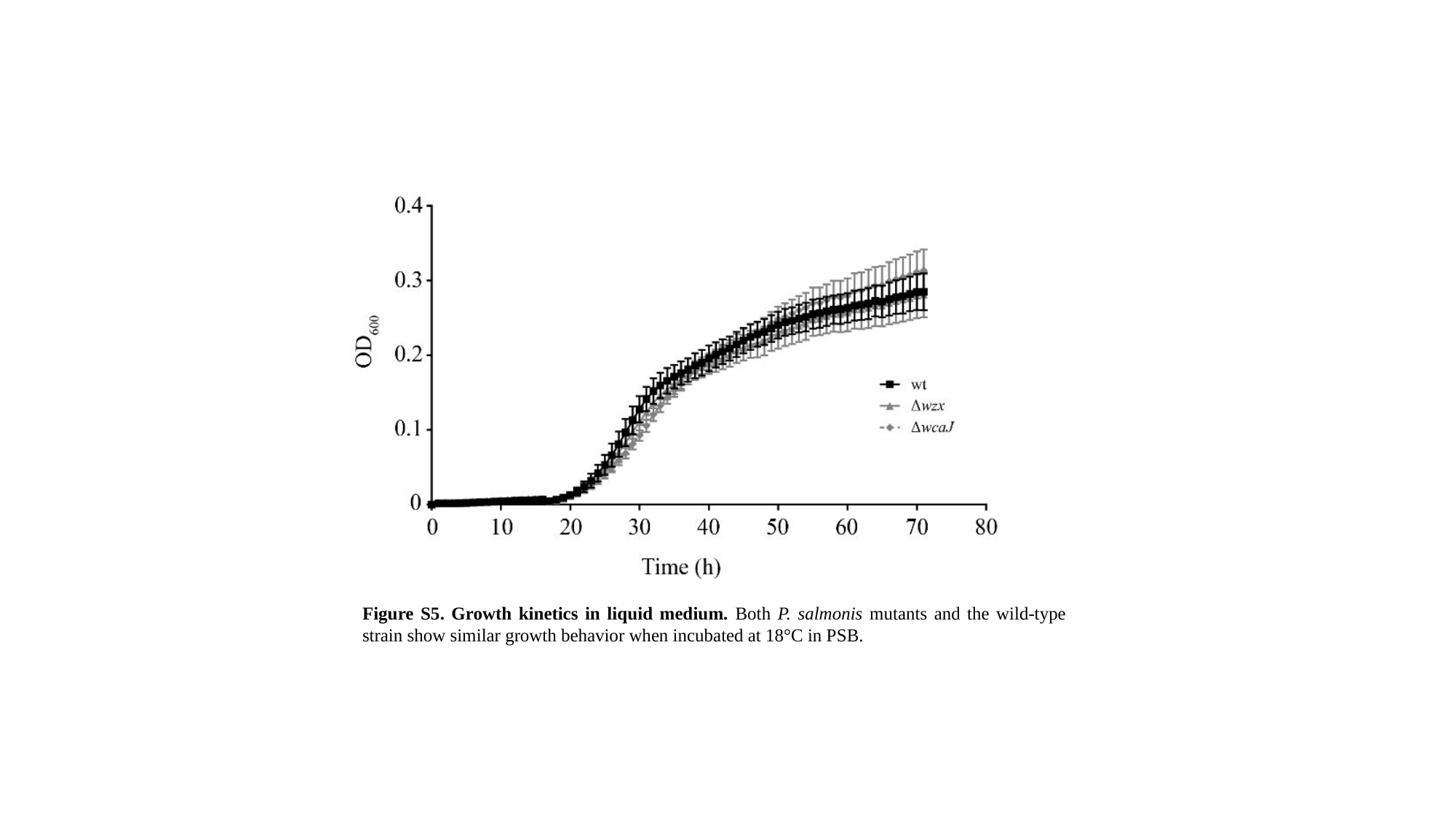

Figure S5. Growth kinetics in liquid medium. Both P. salmonis mutants and the wild-type strain show similar growth behavior when incubated at 18°C in PSB.

## Slide 7
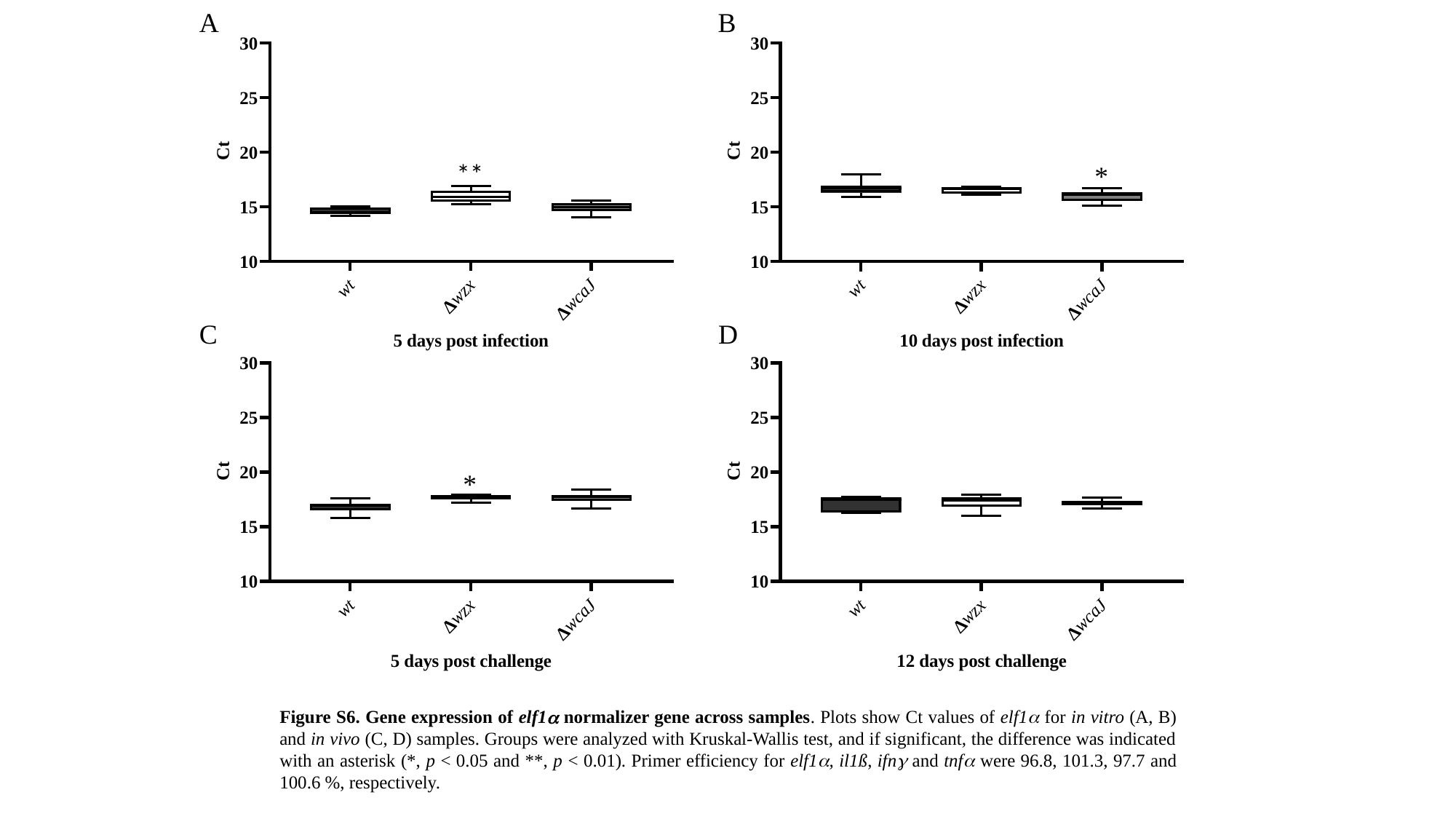

A
B
**
*
C
D
*
Figure S6. Gene expression of elf1α normalizer gene across samples. Plots show Ct values of elf1α for in vitro (A, B) and in vivo (C, D) samples. Groups were analyzed with Kruskal-Wallis test, and if significant, the difference was indicated with an asterisk (*, p < 0.05 and **, p < 0.01). Primer efficiency for elf1α, il1ß, ifnγ and tnfα were 96.8, 101.3, 97.7 and 100.6 %, respectively.

## Slide 8
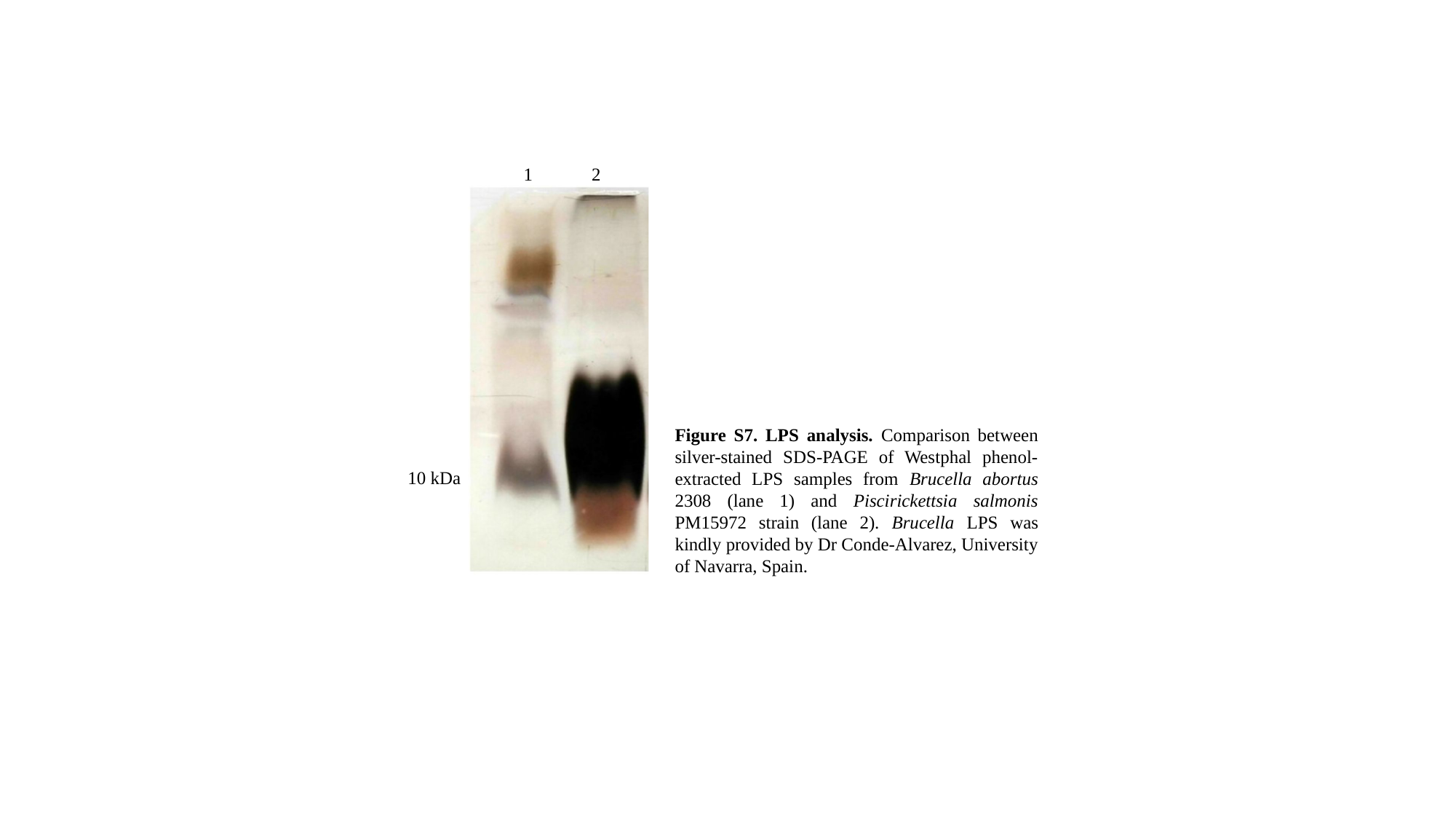

1 2
10 kDa
Figure S7. LPS analysis. Comparison between silver-stained SDS-PAGE of Westphal phenol-extracted LPS samples from Brucella abortus 2308 (lane 1) and Piscirickettsia salmonis PM15972 strain (lane 2). Brucella LPS was kindly provided by Dr Conde-Alvarez, University of Navarra, Spain.
